# Supplementary material for: Detection of CRISPR‒Cas and type I R–M systems in Klebsiella pneumoniae of human and animal origins and their relationship to antibiotic resistance and virulence
Source: Microbiol Spectr. 2024 Dec 19;13(2):e00009-24. doi: 10.1128/spectrum.00009-24 (PMC11792477; doi:10.1128/spectrum.00009-24)
Supplement: Table S1 — Plasmids and phages homologous to the spacer sequences of 144 CRISPR-Cas system positive strains. [file spectrum.00009-24-s0002.docx]

**Table S1** Plasmids and phages homologous to the spacer sequences of 144 CRISPR-Cas system positive strains

| Spacers | Plasmids | Percent identity | Assession no. | Phages | Percent identity | Assession no. |
| --- | --- | --- | --- | --- | --- | --- |
| k37 | p2020S09-154-727k | 100% | CP129873.1 |  |  |  |
| K38 | / |  |  |  |  |  |
| K42 | / |  |  |  |  |  |
| K53 | / |  |  |  |  |  |
| K54 | / |  |  |  |  |  |
| K56 |  |  |  | Enterobacter phage Arya | 100% | NC_031048.1 |
| K59 | / |  |  |  |  |  |
| K60 | / |  |  |  |  |  |
| K62 | / |  |  |  |  |  |
| K63 | / |  |  |  |  |  |
| K64 | / |  |  |  |  |  |
| K65 | / |  |  |  |  |  |
| K68 |  |  |  | Enterobacter phage Arya | 100% | NC_031048.1 |
| K70 | / |  |  |  |  |  |
| K72 |  |  |  | Klebsiella phage KP13-16 | 96.97% | OP617741.1 |
| K88 | / |  |  |  |  |  |
| K89 | / |  |  |  |  |  |
| K90 | / |  |  |  |  |  |
| K91 | / |  |  |  |  |  |
| K92 | / |  |  |  |  |  |
| K93 |  |  |  | Shigella phage DS8 | 95.35% | NC_054650.1 |
| K94 | / |  |  |  |  |  |
| K95 | / |  |  |  |  |  |
| K97 | / |  |  |  |  |  |
| K98 | / |  |  |  |  |  |
| K100 | / |  |  |  |  |  |
| K102 | / |  |  |  |  |  |
| K103 |  |  |  | Klebsiella phage vB_KpM_FBKp34 | 95.45% | MW394390.1 |
| K105 | / |  |  |  |  |  |
| K107 | / |  |  |  |  |  |
| K108 | / |  |  |  |  |  |
| K109 | P2018N21-088-219k | 100% | CP219749.1 |  |  |  |
| K110 | / |  |  |  |  |  |
| K111 | / |  |  |  |  |  |
| K112 |  |  |  | Klebsiella phage KP13-16 | 95.45% | OP617741.1 |
| K113 | / |  |  |  |  |  |
| K114 | / |  |  |  |  |  |
| K117 |  |  |  | Enterobacter phage Arya | 100% | NC_031048.1 |
| K118 | / |  |  |  |  |  |
| K119 | / |  |  |  |  |  |
| K122 | / |  |  |  |  |  |
| K123 | / |  |  |  |  |  |
| K124 | / |  |  |  |  |  |
| K125 | pKa_200023_1 | 100% | CP084875.1 |  |  |  |
| K126 | / |  |  |  |  |  |
| K127 | / |  |  |  |  |  |
| K129 | / |  |  |  |  |  |
| K130 | / |  |  |  |  |  |
| K135 | / |  |  |  |  |  |
| K141 | / |  |  |  |  |  |
| K143 | / |  |  |  |  |  |
| K152 | / |  |  |  |  |  |
| K168 | / |  |  |  |  |  |
| K183 | / |  |  |  |  |  |
| K184 | pEC105227S | 100% | CP110987.1 |  |  |  |
| K186 | / |  |  |  |  |  |
| K188 |  |  |  | vB_Kpn_Chronis | 100% | MN013086.1 |
| K191 |  |  |  | Klebsiella phage KP13-16 | 96.88% | OP617741.1 |
| K206 | / |  |  |  |  |  |
| K215 | / |  |  |  |  |  |
| K217 | pKPC2_KP26 | 97.37% | CP133745.1 |  |  |  |
| K218 | / |  |  |  |  |  |
| K237 | pEC105227S | 100% | CP110987.1 |  |  |  |
| K238 | / |  |  |  |  |  |
| K240 | / |  |  |  |  |  |
| K242 | / |  |  |  |  |  |
| K243 | / |  |  |  |  |  |
| K246 | / |  |  |  |  |  |
| K254 |  |  |  | vB_Kpn_Chronis | 100% | MN013086.1 |
| K256 | / |  |  |  |  |  |
| K263 | / |  |  |  |  |  |
| K264 | pSID2 | 100% | CP066513.1 |  |  |  |
| K266 | p1_130119 | 100% | CP127232.1 |  |  |  |
| K270 |  |  |  | Klebsiella phage KP13-16 | 96.88% | OP617741.1 |
| K273 | pEC105227S | 100% | CP110987.1 |  |  |  |
| K276 | / |  |  |  |  |  |
| K279 |  |  |  | Vibro phage pYD38-A | 100% | NC_021534.1 |
| K280 | / |  |  |  |  |  |
| K284 | / |  |  |  |  |  |
| K291 |  |  |  | Enterobacter phage Arya | 100% | NC_031048.1 |
| K292 |  |  |  | Klebsiella phage vB_KpM_FBKp34 | 95.45% | MW394390.1 |
| K294 | / |  |  |  |  |  |
| K296 |  |  |  | Klebsiella phage KP13-16 | 96.88% | OP617741.1 |
| K299 | / |  |  |  |  |  |
| K301 |  |  |  | Klebsiella phage vB_KpM_FBKp34 | 95.45% | MW394390.1 |
| K303 |  |  |  | Enterobacter phage Arya | 100% | NC_031048.1 |
| K304 | / |  |  |  |  |  |
| K305 | / |  |  |  |  |  |
| K307 |  |  |  | Enterobacter phage Arya | 100% | NC_031048.1 |
| K311 | / |  |  |  |  |  |
| K313 | / |  |  |  |  |  |
| K327 | pEC105227S | 100% | CP110987.1 |  |  |  |
| K328 |  |  |  | Enterobacter phage Arya | 100% | NC_031048.1 |
| K330 | / |  |  |  |  |  |
| K333 | pAMA1416 | 100% | MG462728.1 |  |  |  |
| K340 | / |  |  |  |  |  |
| K346 |  |  |  | Klebsiella phage KP13-16 | 96.88% | OP617741.1 |
| K347 |  |  |  | Enterobacter phage Arya | 100% | NC_031048.1 |
| K348 |  |  |  | Enterobacter phage Arya | 100% | NC_031048.1 |
| K350 | / |  |  |  |  |  |
| K352 | pEC105227S | 100% | CP110987.1 |  |  |  |
| K353 |  |  |  | Enterobacter phage Arya | 100% | NC_031048.1 |
| K357 | / |  |  |  |  |  |
| K367 | / |  |  |  |  |  |
| K368 | / |  |  |  |  |  |
| K369 | / |  |  |  |  |  |
| K370 | / |  |  |  |  |  |
| K371 | / |  |  |  |  |  |
| K373 | / |  |  |  |  |  |
| K383 | / |  |  |  |  |  |
| K384 | / |  |  |  |  |  |
| K389 |  |  |  | Enterobacter phage Arya | 100% | NC_031048.1 |
| K404 | / |  |  |  |  |  |
| K430 | pEC105227S | 100% | CP110987.1 |  |  |  |
| K434 | pEC105227S | 100% | CP110987.1 |  |  |  |
| K439 | / |  |  |  |  |  |
| K440 | / |  |  |  |  |  |
| K444 | pEC105227S | 100% | CP110987.1 |  |  |  |
| K445 | pEC105227S | 100% | CP110987.1 |  |  |  |
| K446 |  |  |  | Enterobacter phage Arya | 100% | NC_031048.1 |
| K451 | / |  |  |  |  |  |
| K454 | / |  |  |  |  |  |
| K456 | / |  |  |  |  |  |
| K484 | pEC105227S | 100% | CP110987.1 |  |  |  |
| K491 | / |  |  |  |  |  |
| K493 | pSID2 | 100% | CP066513.1 |  |  |  |
| K494 | pKPC2_CFR867 | 100% | CP151861.1 |  |  |  |
| K499 | / |  |  |  |  |  |
| K503 | pCY-CTXM-15 | 82.14% | OV141394.1 |  |  |  |
| K507 | pEC105227S | 100% | CP110987.1 |  |  |  |
| K508 | Pec422_1 | 81.25% | CP018961.1 |  |  |  |
| K513 | / |  |  |  |  |  |
| K514 | / |  |  |  |  |  |
| K518 | pSYCC2_tmex_279k | 81.20% | CP113186.1 |  |  |  |
| K519 | p2020S09-154-727k | 100% | CP129873.1 | Klebsiella phage vB_KpM_FBKp34 | 95.45% | MW394390.1 |
| K521 | / |  |  |  |  |  |
| K522 | pEC105227S | 100% | CP110987.1 |  |  |  |
| K526 | / |  |  |  |  |  |
| K527 | / |  |  |  |  |  |
| K529 | / |  |  |  |  |  |
| K530 | / |  |  |  |  |  |
| K531 | pEC105227S | 100% | CP110987.1 |  |  |  |
| K532 | / |  |  |  |  |  |
| K533 | / |  |  |  |  |  |
